# Overcoming barriers to off-patent drug repurposing: a lifecycle-based policy solutions

**DOI:** 10.3389/fphar.2025.1670845

**Published:** 2025-10-24

**Authors:** Mario Garcia-Diaz, David Epstein, Jaime Espin

**Affiliations:** ^1^ Andalusian School of Public Health, Granada, Spain; ^2^ Faculty of Economics and Business, University of Granada, Granada, Spain; ^**3**^ Department of Applied Economics, University of Granada, Granada, Spain; ^4^ Instituto de Investigación Biosanitaria ibs, Granada, Spain; ^5^ CIBER of Epidemiology and Public Health (CIBERESP), Madrid, Spain

**Keywords:** Drug repurposing, off-patent medicines, policy solutions, Barriers, Lifecycle, public Funding, academic research, non-profit organisations

## Abstract

**Introduction:**

Drug repurposing leverages existing medicines to reduce time and costs for exploring new therapeutic indications. However, off-patent medicines do not provide enough private incentives for investment, and prescribing by active ingredient can be a major barrier. Academics and non-profit sponsors also face regulatory barriers.

**Methods:**

This study combined a structured literature review with a questionnaire completed by 25 drug repurposing experts to identify key challenges and policy solutions across the full lifecycle of off-patent drug repurposing.

**Results:**

Public funding is generally available for the early-stage research, where opportunities often arise from off-label uses and real-world evidence. However, limited support for confirmatory clinical trials, combined with the predominance of academics and non-profit sponsors lacking regulatory objectives, reduces the likelihood of successful authorisation to below 30%. Policy solutions include prioritising research into rare diseases and other conditions with unmet health needs, and call for a more prominent role of governments in public investment and in reinforcement of public–private partnerships. Substitution in community pharmacies remains a major challenge, which may be addressed through indication-based differentiation and enhanced dispensing controls. Some innovative public intervention mechanisms, such as Social Impact Bonds and the InterVentional PharmacoEconomics proposal, offer potential, although real-world experience is still limited.

**Conclusion:**

Off-patent drug repurposing faces persistent financial and regulatory barriers that require a central role from academics and non-profit sponsors, supported by governmental engagement throughout the lifecycle. Although steps have been taken in recent years, efforts remain insufficient, and structural and political barriers continue to hinder broader implementation.

## 1 Introduction

Drug repurposing is the process of identifying a new use for a medicine or active substance for an indication that is outside the original target indications (Commission Expert Group on, 2025; van der Pol et al., 2021). Drug repurposing has several advantages over new drug development (Table 1) (van der Pol et al., 2021; Drug Discovery from Technology Networks, 2022). The pharmacokinetics, pharmacodynamics, preclinical toxicology and safety profile of the medicine in the new indication may be known if the dose, duration of treatment and target population are comparable. Dose selection take into account existing knowledge (van der Pol et al., 2021; Kesselheim and Liu, 2024; L et al., 2024; de Visser et al., 2024). Thus, large and expensive preclinical studies and Phase I and II clinical studies may not be necessary (van der Pol et al., 2021; Kesselheim and Liu, 2024). In addition, drug repurposers exploit the structure-activity relationships of approved medicines to target previously unknown pathways relevant to new disease indications through compound identification strategies such as *in silico* screening (computational with artificial intelligence (AI)) and activity-based screening (biological), as well as sophisticated *in vitro* models (Drug Discovery from Technology Networks, 2022). This may lead to faster development with benefits to patients. For example, artesunate is a medicine used as an anti-malarial agent that attracted attention because of the extensive range found with preclinical evidence of anticancer activity, so its potential benefit would be immense in economic and social terms if it were shown to be clinically effective against cancer (Augustin et al., 2015). Finally, many of these medicines are used off-label for the new indication, and this can generate Real-World Evidence (RWE) on their effectiveness that can be used to support applications for marketing authorisation for repurposing (Tan et al., 2023). The COVID-19 pandemic supercharged the advances in drug repurposing, which showed faster development was possible in emergency situations and of great benefit to society (Drug Discovery from Technology Networks, 2022; Kesselheim and Liu, 2024).

**TABLE 1:** Traditional drug discovery vs Drug Repurposing (Adapted from Drug Discovery from Technology Networks: *Drug repurposing: strategies, challenges and successes* (van der Pol et al., 2021)).

Features	Traditional drug discovery	Drug repurposing
Estimated Cost	>2300M€ (Drug Discovery from Technology Networks, 2022)	<460M€ (Kesselheim and Liu, 2024; L et al., 2024)
Time to the clinic	10–15 years (de Visser et al., 2024; Augustin et al., 2015)	3–12 years (Tan et al., 2023)
Failure Rate (a)	90%–95% (Augustin et al., 2015)	25%–70% (Tan et al., 2023)
Preclinical research	1. Target validation 2. Compound screening 3. Lead optimisation (SAR, drug-like properties, solubility, etc.)	1. *In silico* screening 2. Activity-based screening
Clinical trial requirements	Phases I-III	Phase II (depends) and III

(a) Success = Commercial authorisation in the new indication.

Furthermore, off-label use can be disadvantageous for patients, clinicians and health systems. Obtaining regulatory approval in these indications might reduce clinical uncertainty, improve equity of access, increase transparency and regulatory control, make the overall market for a given active pharmaceutical ingredient more sustainable, and give greater stimulus to research and development (R&D).

Obtaining marketing authorisation of an existing medicine for a new indication requires investment in clinical studies and can be costly and time consuming (van der Pol et al., 2021; de Visser et al., 2024). Nevertheless, while the medicine is on-patent or enjoys data or market protections, the rewards for achieving approval in multiple indications can also be large for the patent holder. There are many examples where Marketing Authorisation Holders (MAH) have developed blockbuster medicines across multiple indications, for example, Humira (adalimumab), Opdivo (nivolumab), Keytruda (pembrolizumab) or Dupixent (duplimab) (Griffiths et al., 2023).

However, when commercial protection ends and generic or biosimilar versions enter the market, the economics of repurposing becomes very different. This article focuses on the repurposing of a commercially authorised medicine after data and patent protection has ended.

Despite the potential benefits, entities considering repurposing an off-patent medicine face numerous financial and regulatory challenges.

Generic or biosimilar medicines no longer have patent protection and are therefore subject to a competitive market (van der Pol et al., 2021; Kesselheim and Liu, 2024; Breckenridge and Jacob, 2019; Bloom, 2015; Scholte et al., 2025). If a potential commercial developer cannot obtain further years of market protection, then they may not be able to charge higher prices for the “second medical use” to recover the costs of the clinical studies and fees for regulatory authorisation (van der Pol et al., 2021; Kesselheim and Liu, 2024; de Visser et al., 2024; Breckenridge and Jacob, 2019; Bloom, 2015; Scholte et al., 2025). If there are several generic or biosimilar manufacturers, there may arise a “free rider “dilemma, whereby no firm wants to undergo the expense of investigation into a new indication if all competitors can benefit from the results without contributing to the cost. If doctors can prescribe the medicine “off-label” in an unauthorized indication, this may undercut incentives to undertake the investment to obtain marketing authorisation (Federati on of American Scientists, 2025).

Where there is a lack of interest by commercial entities, research on this type of repurposing may be carried out by non-profit entities, such as academic researchers (AcaRes) and non-governmental organisations (NGOs). (van der Pol et al., 2021; de Visser et al., 2024; Bloom, 2015; Scholte et al., 2025). Nevertheless, there are barriers in practice. There is a lack of public funding through public agencies or governments to conduct drug repurposing trials. In general, there is low visibility and awareness of drug repurposing in the existing regulatory framework and in the drug development community (Commission Expert Group on, 2025; van der Pol et al., 2021; Kesselheim and Liu, 2024). The Safe and Timely Access to Medicines for Patients (STAMP) group found that, despite having high capacities to generate and analyse data in clinical trials, the main limitations for AcaRes/NGOs considering repurposing projects was the lack of knowledge of the regulatory pathways and requirements, as well as the lack of resources to legally carry out the role of an MAH for commercial approval and post-marketing responsibilities (Commission Expert Group on, 2025; van der Pol et al., 2021). Market access for the new indication might also require health technology assessment which adds to the complexity and cost for potential developers. (Breckenridge and Jacob, 2019). While in principle drug repurposing can borrow from existing knowledge and off-label data, in practice there are limits to how far this can be taken. Removing barriers to access to data from failed trials might facilitate drug repurposing opportunities (Commission Expert Group on, 2025; Drug Discovery from Technology Networks, 2022; Breckenridge and Jacob, 2019).

This research analyses the barriers and potential solutions to support drug repurposing projects, including the role of different funding mechanisms. In addition, this research provides examples of real cases throughout the drug repurposing lifecycle and recommends policy solutions to encourage and implement drug repurposing projects.

## 2 Methods

The work was based on (1) a narrative literature review and (2) a questionnaire-based survey to experts who have knowledge and/or experience in any part of the life cycle of drug repurposing.

### 2.1 Narrative literature review

#### 2.1.1 Scope and exclusion criteria

Based on the research objectives, the narrative literature review included the most relevant articles containing the following information: 1) regulatory solutions and 2) pricing and reimbursement (P&R) solutions for repurposed medicines, 3) government support for R&D 4) support AcaRes/NGOs to conduct drug repurposing research, or (5) public intervention mechanisms to carry out drug repurposing projects. In addition, we excluded articles prior to 2011, articles that were not in English or Spanish, articles that did not directly address the objectives of the review, and articles that, throughout the article, did not add anything new to the information already collected.

#### 2.1.2 Search technique

An academic literature search was conducted on PubMed and Embase using a search strategy based on the chosen scope (Supplementary Material 1). Considering this scope and the exclusion criteria for article screening, a flowchart was developed (Supplementary Material 1). The Rayyan software (https://www.rayyan.ai/) was used for the review management. An exploratory targeted search of grey literature was conducted using Google to identify mechanisms for public intervention in drug repurposing and websites for government-sponsored collaborative drug repurposing projects (Supplementary Material 1). Given the exploratory nature, records were not logged with the same PRISMA-style counts as the academic search. The technique used to carry out the grey literature search was the ‘snowballing’ technique (Rejon-Parrilla et al., 2022). This search ended on 26 March 2025.

### 2.2 Questionnaire

#### 2.2.1 Questionnaire design

The questionnaire was designed following a review of the literature and preliminary interviews with two experts in repurposing, first, a representative of the Spanish Ministry of Health and second, a leading preclinical research scientist. A draft of the questionnaire was piloted with a third expert in pharmaceutical policy to improve the clarity and appropriateness of the questions.

#### 2.2.2 Content of the questionnaire

The questionnaire is shown in English and Spanish in Supplementary Material 2. It begins with an introduction in which the objectives are explained to the respondent. The socio-demographic profile questions include name, surname, age, years of experience and sector of professional activity and the respondent’s experience in repurposing. There are 26 further questions, divided into the following seven blocks, which broadly represent the phases of a repurposing life cycle: (1) Concept (Drug Repurposing), (2) Primary research, (3) Funding of R&D for repurposing, (4) Regulatory aspects of drug repurposing, (5) Price and financing of repurposed medicines, (6) Role of public bodies in drug repurposing and (7) Open questions on real cases. Regarding the type of questions, two of the questions included were Likert-type scales from one to five, four questions were binary (Yes/No), three questions were open-ended text, and the remaining 17 questions asked respondents to select their preferred response(s) from options based on our literature search. 10 of these allowed more than one response. For most questions, a comment box was provided if respondents wished to explain theiranswer or provide nuance. *Selection of experts*.

We surveyed experts who had worked on or had expert knowledge of least one of the phases of the life cycle of off-patent drug repurposing. We began with web searches (Google) of NGOs, projects and research consortia dedicated to drug repurposing, and actors recommended to us by healthcare experts. 17 preliminary candidates were contacted by email, asking if they could participate or recommend colleagues. This email was answered by 11 of these preliminary contacts, who provided 42 people to whom we sent the questionnaire. The selected experts were categorised into six profiles that cover the stages and actors involved: (1) Pharmaceutical industry, (2) Academic research, (3) Health-related NGO, (4) Hospital manager, (5) Regulatory agency and (6) Policymaker.

#### 2.2.3 Application of the questionnaire

The questionnaire was sent by email to each individual expert. In this email, each expert was informed about the questionnaire, the research objectives and the question of their choice as the target expert, and the questionnaire was attached as a Word document^®^. Subsequently, two reminders were sent to experts who had not yet responded.

#### 2.2.4 Analysis of the results

For the quantitative (categorical) responses, a descriptive analysis was carried out using cross-frequency tables. For questions and statements in the qualitative (text) format, textual analysis was used to discern key themes and relevant patterns emergent from them. The responses, both categorical and non-categorical qualitative responses, were collected using an Excel spreadsheet^®^. Categorical data were analysed using Stata^®^ software.

## 3 Results

### 3.1 Narrative literature review

Relevant results are presented based on the research objectives for the 25 articles selected in the narrative literature review (Supplementary Materials 1 and 3).

#### 3.1.1 Regulatory solutions

Several studies consider using regulatory approval as a route to gaining market exclusivity for a new indication, and thereby the possibility of recovering the cost of R&D through higher prices. EU rules allow medicines that qualify for “orphan drug status” to obtain 10 years of market exclusivity, as well as facilities for faster development of the repurposed generic medicine (van der Pol et al., 2021; Pushpakom et al., 2019; Hernandez et al., 2017; Verbaanderd et al., 2019). In the US, in the case of rare or complex diseases, there is also the possibility of obtaining the ‘speciality drug’ designation, which allows high prices (Hernandez et al., 2017).

Some papers discussed strategies for applying for a patent on a new repurposed medicine. These strategies included the development of new formulations, dosage forms, or new derivatives with a similar therapeutic effect would maximise the chances of patentability (Pushpakom et al., 2019).

2 articles in support of drug repurposing were included in 2023 in the draft European Pharmaceutical Strategy (EPS), which aims to provide a common framework for EU Member States (de Visser et al., 2024; Scholte et al., 2025). Article 48 addresses non-profit entities, which could submit evidence for a new therapeutic indication for an authorised medicinal product that is expected to cover an unmet medical need (de Visser et al., 2024; Scholte et al., 2025). In case of a favourable outcome by European Medicines Agency (EMA), MAHs will be required to update the product with the new indication (de Visser et al., 2024; Scholte et al., 2025). Article 84 concerns repurposed medicines that have not previously benefited from regulatory exclusivity or for which 25 years have passed since the marketing authorisation was approved. In these cases, 4 years of data exclusivity may be granted for data provided by studies showing significant clinical benefit for the new indication (de Visser et al., 2024; Scholte et al., 2025).

In the US, the “505(b) (van der Pol et al., 2021) pathway” allows any sponsor to reference safety and effectiveness data from previous approvals and existing data published in the literature that meet regulatory standards, once their exclusivity has ended, with the aim of obtaining a new indication (Hernandez et al., 2017; Crittenden et al., 2024). Currently, this sponsor may be a non-manufacturer collaborating with the current manufacturer of the medicine, but the private company has to be the MAH for the new indication and the entity responsible for distributing the new product with the new indication (Hernandez et al., 2017; Crittenden et al., 2024). Aiming to overcome the lack of incentives for private involvement, the 505(b) (van der Pol et al., 2021) pathway allows a ‘labelling-only’ extension for non-manufacturers who want to apply for approval of a new indication for repurposed generic medicines through an approach that is not linked to a specific product made by a specific manufacturer. Thus, this new indication could be associated with the originator and all generic versions of the medicine (Crittenden et al., 2024). That said, this pathway is only for well-established, commercially available small molecule generic medicines (Crittenden et al., 2024).

Several papers consider regulatory support programmes for AcaRes/NGOs that address problems in the commercial authorisation process for off-patent repurposed medicines. The EMA has developed, through a proposal from a STAMP expert group, a pilot programme with the objective of supporting AcaRes/NGOs in generating sufficient evidence to carry out off-patent drug repurposing of a generic or biosimilar (Commission Expert Group on, 2025; Verbaanderd et al., 2019; Asker-Hagelberg et al., 2021; Jonker et al., 2024). The objective of this proposal is to provide a visible support framework to the NGO stakeholder (*Champion*) by offering scientific advice at an early-stage to facilitate the collection and generation of data according to regulatory standards for a new therapeutic use (Commission Expert Group on, 2025; Verbaanderd et al., 2019; Asker-Hagelberg et al., 2021; Jonker et al., 2024). In this process, the *Champion* will rely on the Scientific Advice (SA) to present the best scientific data to the MAH to encourage it to take the appropriate route, adapting to the enforceable requirements, and apply for a marketing authorisation for a new indication (Commission Expert Group on, 2025). An equivalent programme in the United Kingdom is called The UK Innovative Licensing and Access Pathway (Liddicoat et al., 2024).

#### 3.1.2 Government support for R&D

Some authors argue that the government should provide financial support for repurposing R&D where there are large expected public health benefits but few commercial incentives (van der Pol et al., 2021). Options might include prizes, tax incentives or special research funds to generic producers or small and medium-sized enterprises (SMEs) or academic researchers (Verbaanderd et al., 2019).

Other literature highlights the role of government-sponsored collaborative drug repurposing networks (Table 2).

**TABLE 2:** Government-sponsored collaborative drug repurposing networks.

Program name	Program type	Program members	Operation
UK NHS Medicines Repurposing Programme (Griffiths et al., 2023; DiMasi et al., 2016; Pushpakom et al., 2019; England, NHS, 2022)	Public programme	National agencies, NHS clinicians, charities, patient representatives, pharmaceutical companies	Aims to identify and develop repurposing opportunities for prioritised medicines. It funds research and commissions generic manufacturers for licensing. Medicine inclusion is based on multi-stakeholder criteria
Discovering New Therapeutic Uses for Existing Molecules (US) (Kesselheim and Liu, 2024; DiMasi et al., 2016; Nosengo, 2016; Paul et al., 2010; BIO, 2021; Frail et al., 2015)	Public programme	National Center for Advancing Translational Studies (NCATS)	Provides preclinical support and research grants for phase 1–2 studies for repurposing approved molecules. Phase 3 funding is limited to rare or neglected diseases
CURE Drug Repurposing Collaboratory (CDRC) (US) (DiMasi et al., 2016; Nosengo, 2016)	Public–Private Partnership (PPP)	FDA, NCATS, Critical Path Institute	An online platform (https://cure.ncats.io/home) allowing healthcare providers to report and search for new uses of existing medicines. Data are collected from RWE and literature reviews
Belgian Healthcare Knowledge Centre (KCE) (Roessler et al., 2021; KCE, 2024)	Public programme	KCE (independent public body)	Selects and funds large non-commercial RCTs in repurposing, based on strong scientific evidence and expected return on investment
FDA Project Renewal & MODERN Labeling Act (US) (Nosengo, 2016; Breckenridge and Jacob, 2019)	Public programmes	FDA and other federal institutions	Repurposing initiatives with a prominent role played by the public sector

Source: Own elaboration.

#### 3.1.3 Pricing and reimbursement (P&R) solutions

Several papers highlight the problems in the P&R phase and possible solutions. Second-use medical patents (providing the potential for higher prices in the new indication) are feasible in some countries in certain situations (see “Regulatory Solutions”), but problems can arise where the original formulation and the dosage forms are retained (de Visser et al., 2024; Pushpakom et al., 2019; Breckenridge and Jacob, 2019; Crittenden et al., 2024). For example, in many systems, a doctor prescribes by active ingredient, and the pharmacist dispenses the lowest cost version of that prescription (pharmacy substitution). If the pharmacist has no knowledge of the intended use or is reimbursed at a fixed level regardless of whether the dispensed medicine is a branded or generic version, then the manufacturer will not be able to recover R&D costs through higher prices (de Visser et al., 2024; Pushpakom et al., 2019; Breckenridge and Jacob, 2019; Crittenden et al., 2024).

A potential solution to the dispensing problem is to ensure, in a legal manner or across differential pricing system, that indication information is provided on all prescriptions alongside the non-proprietary name (de Visser et al., 2024; Breckenridge and Jacob, 2019; Verbaanderd et al., 2019). Thus, the pharmacy will know whether the prescription corresponds to the branded version (with the new indication) or whether it is appropriate to dispense the generic/biosimilar version (de Visser et al., 2024; Breckenridge and Jacob, 2019). In addition, national governments could ensure that reimbursement will be adapted at different levels depending on the indication for which it is to be used and thus differentiate between on-patent and off-patent uses (de Visser et al., 2024; Breckenridge and Jacob, 2019). This measure aims to provide greater market predictability for investors (de Visser et al., 2024).

#### 3.1.4 Mechanisms for financing drug repurposing projects through public intervention

##### 3.1.4.1 Social Impact Bonds

Some authors have considered mechanisms to bring in a wider community of stakeholders and investors. One option is the Social Impact Bond (SIB) (Bloom, 2015). This is a collaboration between three partners: an impact investor (often a private philanthropic organisation or individual) who can provide upfront funding at the risk they will not recover their capital or make a return; an implementing organisation to deliver the research programme (often NGO or charities); and an outcomes funder (for example, a government or health commissioner) who will pay back the investor if and when the project achieves the desired outcomes (Bloom, 2015; SSIR, 2024). This mechanism would not require either governments or pharmaceutical companies to provide significant upfront funding for clinical research (Bloom, 2015; SSIR, 2024). Compared with paying for new medicines developed in the conventional commercial way, there could be net savings to payers (Bloom, 2015; SSIR, 2024). For example, Cures Within Reach (a global NGO), collaborating with impact investors such as the Bridges Social Impact Bond Fund, successfullyrepurposed the generic medicine sirolimus for autoimmune lymphoproliferative syndrome (ALPS) (SSIR, 2024). Sirolimus treatment avoided the need for many hospitalisations and reduced or eliminated other more expensive forms of treatment, resulting in net cost savings to the US healthcare system (SSIR, 2024). Nevertheless, it is challenging to estimate *ex-ante* the likely social impact or predict the cost savings that will be released by the use of repurposed medicines in clinical practice (Verbaanderd et al., 2021).

##### 3.1.4.2 InterVentional PharmacoEconomics rendomised controlled trials + advance market commitment

Several studies comment on a set of proposals known collectively as “InterVentional PharmacoEconomics (IVPE)”. This seeks to provide evidence from Randomised Controlled Trials (RCT) that allows practitioners to decrease prescribing costs while maintaining effectiveness. One strategy could be to replace an expensive standard treatment with a repurposed generic or biosimilar (Federati on of American Scientists, 2025; Kerdemelidis, 2024; Goldstein et al., 2023). The MAH of the standard treatment might not have market incentives to conduct these types of studies. However, from the perspective of the health service payer, if the cost savings from patients taking the low-cost intervention in one arm are sufficiently large, this can mean the RCT will be self-funding. Even if the RCT is not self-funding, but there are expected to be long-term savings, the payer might use prize-type contracts such as Advance Market Commitments (AMCs) to repay the costs of the investigators, in effect committing a fraction of future expected savings to finance the investment. Also, AMCs can provide a financial incentive to obtain regulatory approval that could cost less than the billions of dollars that payers often have to pay for a new patented medicine over the patent lifetime (Federati on of American Scientists, 2025; Kerdemelidis, 2024).

(Federati on of American Scientists, 2025) proposed that the US Congress should mandate that the National Institutes of Health (NIH) and National Center for Advancing Translational Studies (NCATS) work together with payers to pass on a portion of the cost savings from IVPE RCTs and AMCs to create a self-sustaining IVPE + AMC Fund (Federati on of American Scientists, 2025). A limitation of IVPE studies is that they will only work in health systems that have budgetary flexibility to return future savings to finance the research fund and do not suffer from free-rider problems. Hence such iniciatives need to be conducted under large closed systems or single payer systems (Goldstein et al., 2023). Goldstein et al. (2023) suggests the creation of a global consortium of health system payers to conduct IVPE studies (Goldstein et al., 2023). Under the consortium, trials could be scaled up rapidly with a larger patient accrual, and many trials could be conducted simultaneously (Goldstein et al., 2023).

##### 3.1.4.3 Funding via the Best Pharmaceutical for Children Act (BPCA)

One paper considers a legal mechanism in the US through which public funding from the NIH is earmarked for paediatric medicines (Kesselheim and Liu, 2024). One provision of the BPCA is section 409(i)), which provides a direct public funding framework for important medicine studies in children (Kesselheim and Liu, 2024). Under 409(i), the NIH collaborates with the FDA and paediatric preclinical experts to create a prioritised list of therapeutic knowledge gaps in paediatrics, focusing on off-patent medicines (Kesselheim and Liu, 2024). In addition, the BPCA included a provision allowing the NIH to initiate labelling changes with manufacturer compliance (Kesselheim and Liu, 2024). For example, in 2024 the FDA updated the labelling of the antifungal medicine fluconazole for *candida* prophylaxis in preterm infants (Kesselheim and Liu, 2024). So, given that this statutory public funding mechanism is dedicated to a niche market without financial incentives such as off-patent paediatric medicines, an analogous 409(i) programme could be created to focus on repurposing off-patent medicines (Kesselheim and Liu, 2024). A translational research expert group could be convened to select a priority list for repurposing and clinical trials could focus on unmet medical needs (Kesselheim and Liu, 2024).

##### 3.1.4.4 Crowdfunding

Some papers discuss crowdfunding, which allows the contribution of small donations from many people, often non-commercial sponsors, through online platforms. This model would therefore empower patient communities to help drive research in areas important to them (Augustin et al., 2015; Verbaanderd et al., 2021). Crowdfunding can be an interesting model for young investigators and viable for supporting small proof-of-concept trials, thoughthis mechanism is unlikely to provide sufficient funding for large RCTs (Verbaanderd et al., 2021). For example, the NeoART study is an example of a drug repurposing project that raised funds (£54,247) through a crowdfunding campaign on FutSci.com (Augustin et al., 2015; Verbaanderd et al., 2021).

##### 3.1.4.5 Public-private partnerships (PPP)

Academic researchers and disease foundations conduct investigation but often do not have the infrastructure or expertise to take this knowledge forward to develop medicines (Muthyala, 2011). Some authors describe PPP solutions for drug repurposing to address this gap.

The Learning Collaborative is a collaboration that combines the disease and network expertise of The Leukaemia & Lymphoma Society (LLS), the cancer drug repurposing and drug development expertise of The University of Kansas Cancer Center (KUCC) and the drug discovery expertise of the NCGC to create a pipeline of new therapies to treat blood cancers (Weir et al., 2025). This collaboration was one of the first NGO/academic initiatives to receive a Cooperative Research and Development Agreement (CRADA) designation, which provides institutional and scientific backing from the NIH, privileged access to public resources, and legal and strategic advantages (Weir et al., 2025). The Learning Collaborative aims to bridge the gap (colloquially known as the “Valley of Death”) between cancer repurposing research, conducted normally by academic researchers, and drug discovery, development and approval, more effectively that any of the component organisations could do alone (Muthyala, 2011; Weir et al., 2025).

The Dutch and Australian governments co-funded the “LoDoCo2” trial through a PPP with several generic pharmaceutical companies to include a new indication for colchicine for cardiovascular disease (de Visser et al., 2024). In this case, this new indication had a dose change to 0.5mg, which, in the case of the US, where no such dose formulation existed, was a monopolistic repurposing (de Visser et al., 2024). Through this situation, the cross-label dispensing barrier was circumvented, so that companies are able to offer the repurposed medicine at a substantially higher price, although certain actions are still needed to ensure access (de Visser et al., 2024). This mechanism would not be relevant in the EU as there are already generics with 0.5 mg doses (de Visser et al., 2024).

### 3.2 Analysis of the expert questionnaire

The questionnaire was answered by 25 experts from different fields (Supplementary Material 4). 12 experts considered themselves very active or exclusively dedicated to drug repurposing, and we categorized this group as the “most involved experts (MIE)”.

#### 3.2.1 Categorical qualitative analysis

The detailed results of the categorical qualitative analysis are summarised in Supplementary Material 5. The most significant findings of the questionnaire are shown below, grouped according to its role in the lifecycle of drug repurposing. Not all experts answered all questions; hence the denominator may be less than N = 25 for some questions.

##### 3.2.1.1 Challenges

The majority of experts highlighted the need for greater economic support and incentives for both AcaRes and NGOs (60%, n = 15/25). 75% (n = 9/12) of the most involved experts considered the main challenge to be the development of incentives for the private sector.

##### 3.2.1.2 Primary research

Experts considered that the identification of new opportunities for repurposing medicines is mainly based on evidence from off-label use (91%, n = 20/22) and RWE studies (77%, n = 17/22). 73% (n = 11/15) considered the probability of success from identification to repurposing to be less than 30%.

##### 3.2.1.3 R&D funding for repurposing

The main financial barriers to R&D identified included the lack of incentives for the private sector (60%, n = 15/25) (mainly supported by MIE) and insufficient public funding (56%, n = 14/25) (mainly supported by AcaRes).

Regarding R&D funding mechanisms for AcaRes/NGOs, 74% (n = 14/19) indicated knowledge of public funding mechanisms from health agencies or programmes and 80% (n = 8/10) of AcaRes/NGOs reported accessing at least one funding mechanisms.

##### 3.2.1.4 Regulatory aspects

The main regulatory challenge for AcaRes/NGOs focused on a lack of knowledge of the process (88%, n = 22/25).

The majority (84%, n = 21/25) of respondents were aware of at least one AcaRes/NGOs regulatory support programme. All academics (n = 8/8), NGOs (n = 3/3) and regulatory experts (n = 12/12) were familiar with the European “Repurposing of Authorised Medicines” programme. Nevertheless, 63% (n = 10/16) considered that its impact has been limited.

Regarding regulatory requirements, 83% (n = 10/12) stated that a Phase 3 clinical trial was requested, but not a Phase 1 or two trial. A majority of 72% (n = 18/25) believed that regulatory approval should be prioritised in cases of unmet medical need.

##### 3.2.1.5 Data exclusivity

MIE (82%, n = 9/11) and price-financing specialists (80%, n = 8/10) believed that offering 4 years of data exclusivity is an effective way to incentivise R&D in repurposing.

##### 3.2.1.6 Role of public bodies

The public sector was seen as a key player in offering regulatory support to AcaRes/NGOs (78%, n = 18/23) and participating in R&D funding (70%, n = 16/23), but 90% (n = 19/21) thought public funding is insufficient. In this regard, 81% (n = 17/21) of the respondents expressed that public investment should play a more active role in this area.

#### 3.2.2 Non-categorical qualitative analysis

This analysis, classified by topic, includes additional comments and answers provided by experts which highlight important issues affecting the drug repurposing lifecycle.

##### 3.2.2.1 Problems and solutions in the P&R phase of drug repurposing

The biggest problem mentioned for the viability of repurposing is the fact that national health systems promote prescribing by active ingredient and policies for the use of lower-priced generics/biosimilars. These policies prevent the application of price differentiation by indication and create problems of pharmacy substitution in those repurposed medicines that maintain the same pharmaceutical form. In addition, the lack of control over dispensing makes it difficult to dispense only the medicine with the authorised indication. Another expert stated that the barrier of including the repurposed medicine in the same reference pricing system as the original indication would have to be overcome.

Experts pointed to price differentiation by indication as a promising strategy, although most of the current national health system are not prepared to apply different prices when the same formulation or route of administration is maintained. Therefore, its implementation needs to be addressed legally and poses certain challenges.

To implement this price differentiation, several strategies were proposed. On the one hand, giving a different name for each indication would generate different marketing numbers, allowing the medicine to be identified and invoiced differently. On the other hand, it was considered essential to improve prescription and auditing systems so that the specific indication for which the medicine is used in each case is recorded and linked to the billing and electronic medical record of each patient.

As a preliminary step, some experts suggested that the monitoring of use by indication could be more feasible in less prevalent pathologies, where it would be possible to carry out control through the pharmacy services and the autonomous communities. Likewise, in the case of medicines for hospital use only, procedures can be more easily adapted to facilitate traceability and control of use by indication, since hospital managers can code the medicine as if it were a different product.

The example of Estonia was also highlighted, where a system of indication-based pricing is already in place. However, it was recognised that this strategy would not be feasible in all countries, especially in those with less digitised health systems. Therefore, some experts concluded that this policy should be adapted to the specificity of each national system and product.

Another expert discussed a regulatory reform that allows for the expansion of indications on the national health authority’s own initiative, in coordination with regulatory agencies and public funders. It was also proposed that incentives should not be limited to new indications only, but should also include new dosage forms, differentiated formulations although it could also face challenges such as the fragmentation of small markets, thus causing sustainability issues and jeopardizing potential price decreases and branding strategies to expand commercial possibilities.

##### 3.2.2.2 Problems and solutions for stimulating research and R&D funding towards commercial approval

One of the main barriers identified by experts in the drug repurposing process was that the data generated in academia are sufficient for scientific publications but do not meet the standards required by regulatory agencies. In addition, review committees evaluating research proposals are often composed of academics with no experience in drug development, which contributes to funding being inefficiently directed more towards basic rather than translational research. As a result, many projects end up being restricted to off-label use.

Currently, many experts recognised public support and grants for early-stage R&D. In this context, risk reduction through preclinical research and clinical trials up to phase 2a is key to facilitating the attraction of additional funding from larger-scale agencies at later stages. Indeed, this is where health NGOs, with public funders, have a strategic role to play. However, even so, most of them do not provide for the necessary steps to reach the market, leaving a gap in both financial and logistical support for large Phase 3 clinical trials, which are essential to obtain approval for new indications.

To overcome this obstacle, many experts believed it is essential to encourage R&D to be more focused on regulatory approval. On the one hand, this means redirecting public funding towards projects that address regulatory requirements at an early-stage and incorporating funds for regulatory processes and later stages of clinical development. To this end, it was proposed that funders should also assess the feasibility of the project´s commercial and regulatory success, as well as its potential to maximise patient access. In this respect, one expert suggested launching public funding campaigns at European level specifically targeted at funding large, randomised, multinational clinical trials. On the other hand, a key solution for the experts to one of the main bottlenecks in the process is to establish collaborations with MAHs in the pharmaceutical industry in search of a new indication on the medicine label.

Finally, it underlines the importance of identifying key therapeutic areas where drug repurposing makes clinical and economic sense, also focusing on unmet medical needs. This approach would reduce the number of projects blocked by market failures, incentivise PPP and increase the likelihood of success.

##### 3.2.2.3 Strategic, regulatory and financial factors in the success of drug repurposing: clinical properties and funding divergences

Experts stated that the success of repurposing depends mainly on the volume and quality of available evidence, the amount of funding, the outcome of clinical efficacy trials and the commitment of the MAH. Despite this, funders’ priorities diverge: private investors focus on return on investment and the likelihood of commercial success, while public funders prioritise public health impact, economic viability and scientific quality.

The areas of clinical management of drug repurposing that experts encountered most are rare diseases and cancer. Both cases, poor access to available treatments and regulatory incentives (for example, Orphan Drug Status for rare diseases) make these pathologies attractive areas for drug repurposing.

On the other hand, experts called for the provision of regulatory mechanisms for accelerated or priority assessment (such as PRIority MEdicines (PRIME) scheme) in the case of drug repurposing, especially to address unmet medical needs. In addition, experts showed that it makes sense to accept RWE data in safety and monitoring trials, although several experts highlighted the need to accept robust studies based on RWE, especially when there is accumulated evidence that could avoid duplication of trials.

##### 3.2.2.4 Alternative forms of financing

Alternative models such as the extrapolation of Paediatric-Use Marketing Authorisation (PUMA) for paediatric indications were mentioned, and it was also proposed to encourage philanthropic initiatives within the private sector guided by ESG principles (for example, in the Access to Medicine Index 2022, although it has not been discussed as a separate topic, Takeda’s initiative in collaboration with Cures Within Reach was in the ReGRoW programme was included) (Supporting researchers in LMICs, 2023). Another option highlighted by one expert was in-kind support, such as the donation of compounds, preclinical services or access to infrastructures such as SciLifeLab (Sweden) or NCATS (US), which can drastically reduce the need for direct funding.

In terms of institutional initiatives, programmes such as REPO4EU, REMEDi4ALL (with a database of funding opportunities), DevelopAKUre, Every Cure, ZonMw, the Gates Foundation (TB experience), and funding from the Centre for Technological Development and Innovation (CDTI) for public-private projects were mentioned (REPO4EU, 2023; REMEDi4ALL, 2023; AKU, 2023; TED Talk Embed, 2023; ZonMw, 2025; Creating an innovative collaboration, 2023).

##### 3.2.2.5 Additional AcaRes/NGOs support programmes for regulatory approval

Several programmes have provided institutional support for drug repurposing, such as the EU’s *Innovative Medicines Initiative (IMI)*, the UK’s *NHS Medicines Repurposing Programme*, and its Australian equivalent. Others include the “Strengthening Training of Academia in Regulatory Sciences” (STARS) project, which promotes regulatory knowledge in academia, the EMA’s innovation office, and the equivalent offices in Belgium (Federal Agency for Medicines and Health Products (FAMHP)) and Germany (*Bundesinstitut für Arzneimittel und Medizinprodukte* (BfArM)) (STARS Proyect, 2022; FAMHP, 2022; BfArM Jahresbericht, 2022). The International Rare Diseases Consortium (*IRDIRC) Drug Repurposing Handbook* was also mentioned (IRDiRC, 2022). It was stated that these programmes have contributed significantly to raising awareness of the value of repurposing and improving its feasibility. However, with the exception of the NHS programme, none of them address the critical step of introducing the medicine on-label (i.e., registering the additional indication).

##### 3.2.2.6 Examples of cases of high public intervention

Flagship examples of publicly supported repurposing mentioned by experts include thalidomide, initially a sedative withdrawn for teratogenic effects, then repurposed for multiple myeloma and leprosy with support from the National Cancer Institute. In addition, remdesivir, developed for Ebola and repurposed for COVID-19 with public funding.

Other cases include the use of bevacizumab in macular degeneration, supported by calls from the Spanish Ministry of Health, and the medicine nitisinone, repurposed thanks to initiatives with public involvement (Estudio, 2022).

## 4 Discussion

**FIGURE 1:**
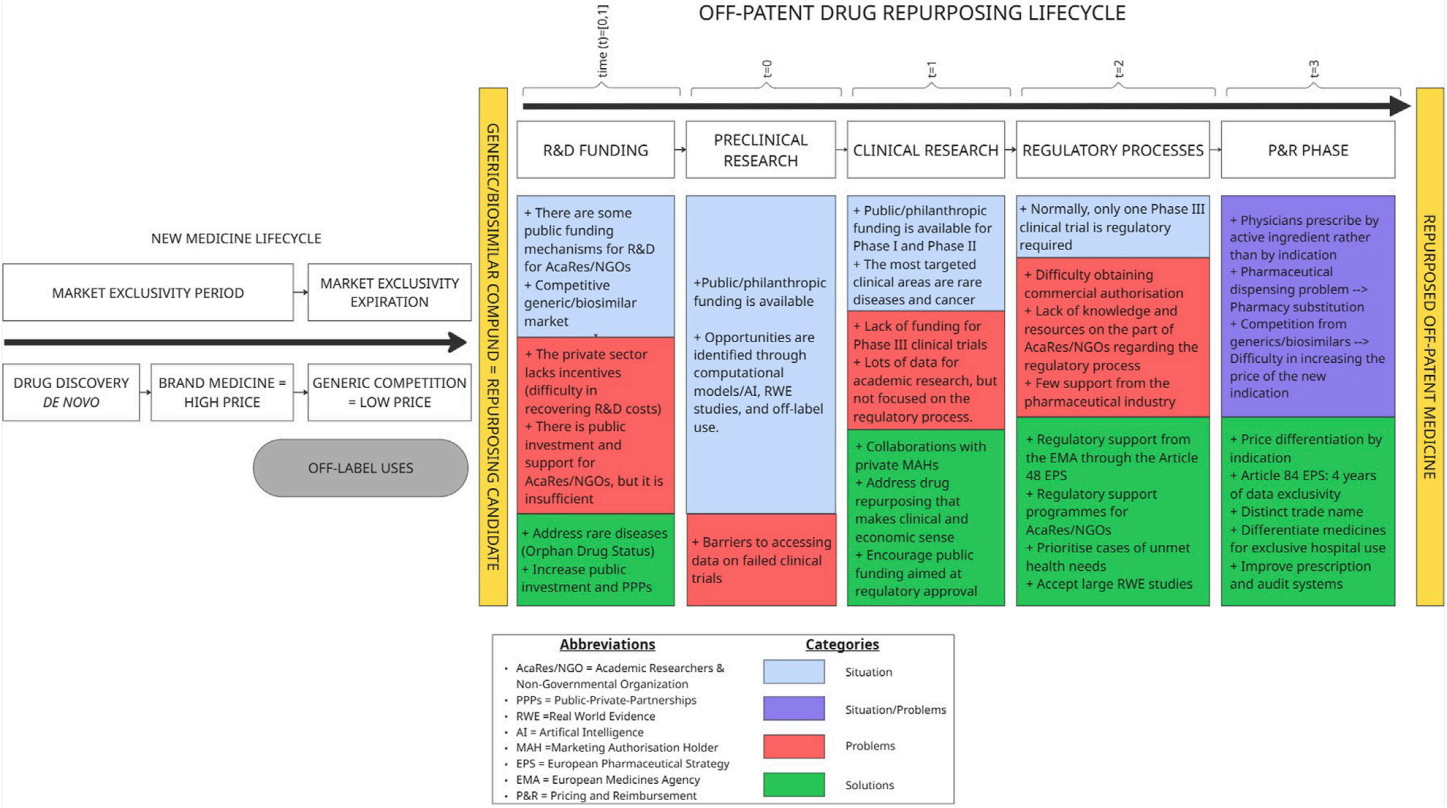
Off-patent drug repurposing lifecycle. Source: Own Elaboration.

To better contextualize our findings, we mapped the scope of regulatory, funding, pricing, and alternative mechanisms supporting drug repurposing across developed countries. As summarized in Table 3, the initiatives identified cover the European Union, the United States, and the United Kingdom, as well as other countries where relevant elements were found (Belgium, Germany, Spain, Sweden, Estonia, and Australia). This mapping highlights the contrast between the more comprehensive frameworks established in the EU, US, and UK, and the more fragmented or absent initiatives in other national contexts (Figure 1).

**TABLE 3:** Geographical scope of policy frameworks and initiatives in drug repurposing.

Category/Scope	EU	US	UK	Other countries
Regulatory solutions	- Orphan Drug Status - Articles 48 & 84 (draft European Pharmaceutical Strategy) - Repurposing of Authorized Medicines (STAMP expert group) - STARS project	- Orphan Drug Status −505(b) (van der Pol et al., 2021) pathway	- Innovative Licensing and Access Pathway	- Belgium: FAMHP - Germany: BfArM - Australia: Medicines Repurposing Programme
Government support for R&D	- REPO4EU - REMEDi4ALL - DevelopAKUre	- Discovering New Therapeutic Uses for Existing Molecules - CURE Drug Repurposing Collaboratory - FDA Project Renewal - MODERN Labeling Act - Gates Foundation	- NHS Medicines Repurposing Programme - Every Cure	- Belgium: Belgian Healthcare Knowledge Centre (KCE), ZonMw - Spain: CDTI
P&R solutions	National health systems promoting prescribing by active ingredient/less digitised health systems			- Estonia (example): indication-based pricing
Alternative mechanisms	- PPPs: Innovative Medicines Initiative (IMI) - Paediatric Use Marketing Authorisation (PUMA)	- Social Impact Bonds: Cures Within Reach - IVPE + AMC (proposal and example in US) - BPCA - PPPs: The Learning Collaborative - In-kind support: NCATS	- Social Impact Bonds: Bridges Fund	- Sweden: SciLifeLab (in-kind support)

Source: Own elaboration.

### 4.1 Main findings

This research identified specific barriers and proposed policy-relevant solutions across the entire lifecycle of drug repurposing projects, from early preclinical research to P&R decisions (Figure 1).

#### 4.1.1 R&D funding

One of the most significant bottlenecks is the lack of commercial incentives to invest in the repurposing of generics/biosimilars. While some public funding mechanisms, PPPs, and crowdfunding initiatives exist to support AcaRes/NGOs, they remain insufficient to bridge the development gap.

Solutions include: targeting orphan drug designation to secure market exclusivity; increasing role of public investment in R&D; and strengthening emerging government-sponsored collaborative drug repurposing projects—such as the NHS Medicines Repurposing Programme—to support AcaRes/NGO-led initiatives as well as broader PPPs.

#### 4.1.2 Preclinical research

In the preclinical phase, opportunities for drug repurposing are identified through computational/AI-based models, RWE studies, and insights derived from off-label use. Much of this work is carried out by AcaRes/NGOs, often benefiting from public and philanthropic funding. However, limited access to failed clinical trial data remains a barrier to identifying repurposing opportunities.

#### 4.1.3 Clinical research

Public funding is commonly available in the early stages of development. However, there is a lack of funding for phase 3 trials aimed at achieving regulatory approval. Furthermore, public funders often prioritise academic output over regulatory pathways. These limitations contribute to the fact that, in most cases, the probability of successfully obtaining a marketing authorisation remains below 30% (see in 3.2.1.2. Primary research).

To address this, experts recommend aligning public research funding with regulatory objectives and encouraging partnerships with MAHs in the pharmaceutical industry. Prioritising clinical areas with unmet medical needs, such as rare diseases and cancer, can increase both clinical relevance and economic feasibility.

#### 4.1.4 Regulatory process

Most drug repurposing projects require at least one phase 3 trial for authorisation, but navigating regulatory procedures is often beyond the capacity of AcaRes/NGOs, given the few participation from the pharmaceutical industry and the limited regulatory support. Due to a lack of resources and knowledge of regulatory processes of this group, there is limited awareness and expertise on how to engage with agencies or act as MAH, in addition to a lack of key funding.

Promising tools include boosting regulatory support for AcaRes/NGOs through the EU’s Article 48 of the EPS, that is able to provide them with a MAH to add a new indication for an unmet medical need, or through programmes like Repurposing Authorised Medicines from EMA, accelerated assessment pathways and greater acceptance of RWE in applications.

#### 4.1.5 P&R phase

The P&R phase is marked by systemic challenges: prescribers usually indicate medicines by active ingredient, enabling pharmacy substitution by generics/biosimilars, and current systems lack mechanisms to recognise new indications in P&R decisions.

Proposed solutions include: establishing indication-based pricing via implementing legal requirements to specify the indication on prescriptions or providing distinct brand names per indication; and using EPS Article 84 to grant 4 years of data exclusivity to the MAH for the new indication. Additional measures include limiting dispensing to hospital-only channels when appropriate, and improving prescription and audit systems.

Likewise, several innovative financing mechanisms have emerged to address the lack of commercial incentives for off-patent drug repurposing. Mechanisms such as Social Impact Bonds (SIBs) allow NGOs to lead trials (for example, the sirolimus trial for paediatric autoimmune diseases) with payment guaranteed only upon success, reducing upfront risk for public funders.

The Interventional Pharmacoeconomics (IVPE) proposal, combined with Advance Market Commitments (AMCs), offers a model to self-fund clinical trials for repurposed generics by leveraging payer cost savings. If the projected savings exceed trial costs, there is no financial risk to funders and later, AMCs would provide incentives for regulatory approval. A proposed IVPE + AMC fund could be sustained by reinvesting those savings.

Public funding frameworks such as the Best Pharmaceuticals for Children Act (BPCA) in the U.S. demonstrate how repurposing for unmet needs can be prioritised through direct NIH support and regulatory collaboration. Similarly, crowdfunding has enabled proof-of-concept studies like NeoART by engaging patients and the public, though its scope remains limited.

Lastly, PPPs offer collaborative platforms for bridging development gaps. Initiatives like the Learning Collaborative and the LoDoCo2 trial show how combining public funding with academic and industry expertise can successfully advance repurposed therapies and navigate funding, regulatory, and access challenges. In this regard, there are PPPs that follow the product development partnership model to advance drug repurposing for global health. Organisations such as DNDi for neglected diseases, GARDP for antimicrobial resistance (AMR) or MMV for malaria; play a critical role in filling R&D gaps that are not commercially attractive, by de-risking development and ensuring equitable access to resulting therapies (Peery, 2022; Kouassi, 2022; Bethencourt-Estrella et al., 2025).

### 4.2 Policy implications

The findings of this study highlight several key policy levers to improve the viability and public value of drug repurposing, especially for off-patent medicines.

First, drug repurposing should be formally recognised as a distinct development pathway within regulatory and funding frameworks. Public policies must offer tailored regulatory support (e.g., EMA’s Repurposing framework, EPS Article 48), opportunities for early scientific and regulatory engagement, and greater acceptance of RWE to facilitate approval processes for new indications.

Second, public research funding should align more closely with regulatory and implementation goals. Funding mechanisms must support not only early-stage studies but also phase III trials and post-marketing requirements.

Third, given that unmet health needs are an interesting area for drug repurposing, it would be useful to establish criteria to guide policymakers who have to decide priority for funding. Factors to consider could include disease burden, disability-adjusted life years lost due to the disease, the affected population, existing alternative options, level of innovation, and so on.

Fourth, non-commercial sponsors (AcaRes, NGOs, and public consortia) require government support to manage development, navigate regulation, and scale implementation. This includes not only technical assistance and shared infrastructure, but also policy and logistical support to establish collaborations with MAHs, which remains one of the key bottlenecks in advancing drug repurposing projects. However, the MAH’s involvement would be limited to requesting a variation of the marketing authorisation in the case of a new indication for an existing formula. In the case of a different formula, the regulatory engagement would be greater.

Fifth, in addition to foster PPPs, alternative financing models must be expanded to attract investment into low-profit but high-impact repurposing projects. Funding schemes such as SIBs and IVPE + AMC offer scalable and outcome-driven solutions. These should be piloted and evaluated across healthcare systems.

Finally, P&R systems must evolve to accommodate the specific challenges of repurposed medicines. Measures such as indication-based pricing, brand name differentiation, and improved prescription traceability can help ensure that new indications are appropriately recognised and reimbursed.

In conclusion, fostering drug repurposing demands coordinated reforms in regulation, funding, pricing and institutional capacity. With appropriate public intervention, repurposing can serve as a cost-effective strategy to expand therapeutic access and address unmet health needs.

### 4.3 Limitations

This study has several limitations that should be considering when interpreting its results. First, the methodological design is based on a narrative review of the literature where, although an exhaustive search was conducted in academic databases and grey literature with inclusion and exclusion criteria, it does not follow a formal systematic protocol as would a systematic review.

Secondly, although the expert questionnaire offers valuable practical insights from different professional profiles, it is based on a somewhat limited sample (n = 25). Furthermore, although they might have international knowledge, these are European experts, which may limit the extrapolation of the findings to regulatory or institutional contexts outside Europe. Also, the expert sample was heterogeneous but unbalanced, with a predominance of participants from academia and hospitals and comparatively fewer from industry and regulatory agencies. This imbalance might affect the generalizability of the findings.

Thirdly, this research is based mainly on qualitative data. Although this research captures a practical view of barriers and solutions, it could introduce subjectivity and variation in interpretation. The lack of quantitative components may limit objectivity.

Finally, although innovative public funding and collaboration mechanisms (e.g., IVPE, SIBs) are analysed, these are still in the pilot phase or have been applied in specific cases, so the derived policy implications should be considered with caution.

### 4.4 Further research

Future research should aim to empirically assess the real-world impact of the policy mechanisms and financing models identified in this study. While the proposed tools show strong theoretical potential, systematic evaluations of their implementation, scalability, and long-term sustainability are still lacking. In this way, incentives such as 4 years of data exclusivity could be compared with other types of regulatory incentives with stronger practical evidence. There is also a need for further exploration of effective collaboration models between AcaRes/NGOs and MAHs, particularly in regulatory submission and market access strategies. Finally, it would also be interesting to research the value of approving an on-label medicine that was being used off-label in the context of drug repurposing, as exemplified by initiatives in the paediatric field such as the Goodman Pediatric Formulations Centre (GPFC), which has worked on transitioning extemporaneous preparations to registered formulations (Litalien et al., 2022).

## 5 Conclusion

This research reveals a set of regulatory and financial barriers that hinder the pharmaceutical industry’s involvement in the repurposing of off-patent medicines. Off-patent drug repurposing has become an area where public sector actors, academic researchers, and non-profit health organisations play a central role. In recent years, governments have begun to support these non-commercial sponsors by introducing public funding mechanisms, regulatory support programmes, and collaboration pathways with MAHs, along with legal reforms such as Articles 48 and 84 of the EPS.

However, the findings suggest that these efforts remain limited, and key political and structural challenges—such as regulatory priorities, funding decisions, and coordination between agencies—persist. The main bottlenecks include the need to align repurposing R&D with regulatory approval goals through increased funding for late-stage clinical trials, incentivising MAH engagement to enhance the likelihood of authorisation, and establishing mechanisms to differentiate the new indication—such as pricing or branding strategies—in order to make commercial participation more viable. Also, focusing drug repurposing efforts on areas of unmet medical need is particularly recommended, as it not only addresses priority health gaps but also increases the likelihood of regulatory support and public funding.

## Data Availability

The original contributions presented in the study are included in the article/Supplementary Material, further inquiries can be directed to the corresponding author.
