# Supplementary material for: Overcoming barriers to off-patent drug repurposing: a lifecycle-based policy solutions
Source: Front Pharmacol. 2025 Oct 24;16:1670845. doi: 10.3389/fphar.2025.1670845 (PMC12592109; doi:10.3389/fphar.2025.1670845)
Supplement: Supplementary file 1 [file DataSheet4.docx]

SUPPLEMENTARY MATERIAL 4: Profile of drug repurposing experts

| Expert | Sector | Gender | Experience | Degree of involvement in Drug Repurposing |
| --- | --- | --- | --- | --- |
| 1 | Health NGO | Female | 10-20 | Very active |
| 2 | Academia | Female | 10-20 | Very active |
| 3 | Academia | Male | 5-10 | Very active |
| 4 | Health NGO | Male | 10-20 | Very active |
| 5 | Hospital | Female | 30-40 | Little active involvement |
| 6 | Hospital | Male | 5-10 | Little active involvement |
| 7 | Academia | Female | 30-40 | Little active involvement |
| 8 | Policymaker | Male | 30-40 | Little active involvement |
| 9 | Policymaker | Male | 10-20 | Little active involvement |
| 10 | Hospital | Female | +40 | Little active involvement |
| 11 | Academia | Male | 30-40 | Little active involvement |
| 12 | Academia | Male | 30-40 | Little active involvement |
| 13 | Academia | Female | 30-40 | Little active involvement |
| 14 | Academia | Female | 10-20 | Little active involvement |
| 15 | Health NGO | Male | 30-40 | Exclusively |
| 16 | Academia | Female | 0-5 | Very active |
| 17 | Hospital | Female | 30-40 | Very active |
| 18 | Hospital | Female | 30-40 | Little active involvement |
| 19 | Hospital | Male | 30-40 | Very active |
| 20 | Hospital | Male | 10-20 | Very active |
| 21 | Pharma industry | Female | 5-10 | Very active |
| 22 | Pharma industry | Male | 30-40 | Little active involvement |
| 23 | Regulatory agency | Female | 5-10 | Very active |
| 24 | Pharma industry | Male | 30-40 | Very active |
| 25 | Pharma industry | Female | 5-10 | Little active involvement |

Source: Own elaboration
