# Supplementary material for: Overcoming barriers to off-patent drug repurposing: a lifecycle-based policy solutions
Source: Front Pharmacol. 2025 Oct 24;16:1670845. doi: 10.3389/fphar.2025.1670845 (PMC12592109; doi:10.3389/fphar.2025.1670845)
Supplement: Supplementary file 2 [file DataSheet3.docx]

SUPPLEMENTARY MATERIAL 3: Papers included in the literature review

|  | Publication Title | Authors and Year | Objectives | Main Findings |
| --- | --- | --- | --- | --- |
| 1 GL | Commission Expert Group on Safe and Timely Access to Medicines for Patients (STAMP) | European Commission (2021) | Provide expert guidance on improving access to medicines in the EU, including drug repurposing. | Recommends coordinated regulatory frameworks and stakeholder collaboration to support safe and timely access to repurposed medicines. |
| 2  A | Drug Repurposing of Generic Drugs: Challenges and the Potential Role for Government | van der Pol et al. (2023) | Analyse the challenges of repurposing generic medicines and explore the government's potential role. | Identifies lack of financial and regulatory incentives as major barriers, proposing public actions to support repurposing. |
| 3  GL | BPCA as a Model for Government-Sponsored Drug Repurposing | Kesselheim & Liu (2024) | Explore BPCA as a model for government-driven drug repurposing. | Identifies lack of financial and regulatory incentives as major barriers, proposing public actions to support repurposing. |
| 4  A | New government drug repurposing programs: Opportunities and uncertainties | Liddicoat et al. (2024) | Describe new government-led drug repurposing programs and assess their challenges. | Highlights new opportunities but also regulatory and operational uncertainties that must be addressed. |
| 5  A | Reliable business case for repurposing of existing medicines requires key changes in government policies | de Visser et al. (2024) | Propose necessary changes in public policies to make drug repurposing business models viable. | Success depends on policy reforms that ensure profitability and market access. |
| 6  A | The wisdom of crowds and the repurposing of artesunate as an anticancer drug | Augustin et al. (2015) | Investigate how collective scientific effort and open data contribute to drug repurposing, using artesunate as a case study. | Demonstrates that community-driven identification of candidates can reveal overlooked therapeutic opportunities and accelerate repurposing. |
| 7  A | Drug repurposing: progress, challenges and recommendations | Pushpakom et al. (2019) | Provide a comprehensive review of the current state of drug repurposing, including scientific, regulatory, and commercial perspectives. | Highlights benefits such as reduced cost and timelines, but also notes major barriers including IP issues, regulatory complexity, and lack of incentives. Recommends enhancing collaboration, funding, and data sharing. |
| 8  A | Overcoming the legal and regulatory barriers to drug repurposing | Breckenridge & Jacob (2019) | Identify and propose solutions to legal and regulatory hurdles limiting drug repurposing efforts. | Advocates for adaptive regulatory frameworks and incentives to support the on-label approval of new uses for existing medicines. |
| 9  A | Creating New Economic Incentives for Repurposing Generic Drugs for Unsolved Diseases Using Social Finance | Bloom (2015) | Explore innovative financing mechanisms to stimulate repurposing of low-cost generics. | Recommends social impact bonds and public-private partnerships as strategies to overcome underfunding in neglected disease areas. |
| 10  A | Revising EU pharmaceutical legislation: will it foster drug repurposing? | Scholte et al. (2025) | Analyse how updates to EU pharma legislation may affect the regulatory environment for repurposing. | Warns that although the proposed reforms offer potential benefits, persistent gaps could undermine repurposing incentives and practical implementation. |
| 11 GL | Leveraging Pharmacoeconomics and Advance Market Commitments to Reduce Healthcare Expenditures | Federation of American Scientists (2023) | Investigate how economic tools can promote cost-effective adoption of repurposed therapies. | Proposes the use of advance market commitments and pharmacoeconomic modelling to align incentives across stakeholders. |
| 12  A | Giving Drugs a Second Chance: Overcoming Regulatory and Financial Hurdles in Repurposing Approved Drugs As Cancer Therapeutics | Hernandez et al. (2017) | Examine the specific regulatory and financial challenges in repurposing already approved medicines for cancer treatment. | Emphasizes limited commercial return from generics and suggests solutions such as public–private partnerships and tailored regulatory pathways. |
| 13  A | On-Label or Off-Label? Overcoming Regulatory and Financial Barriers to Bring Repurposed Medicines to Cancer Patients | Verbaanderd et al. (2019) | Compare the impact of on-label versus off-label use of repurposed medicines in oncology. | Points out limitations of off-label use and advocates for regulatory and economic frameworks to support formal on-label approval of repurposed medicines. |
| 14  A | Pathways for non-manufacturers to drive generic drug repurposing for cancer in the U.S | Crittenden et al. (2024) | Explore how non-commercial actors can lead repurposing of generics for cancer in the U.S. | Discusses regulatory strategies like 505(b)(2) and collaborations with CROs as feasible paths for non-manufacturer-led development. |
| 15  GL | Repurposing of Medicines in the EU: Launch of a Pilot Framework | Asker-Hagelberg et al. (2022) | Present the EU's pilot initiative to formalize and support repurposing efforts. | Describes a collaborative framework engaging regulators, industry and academia to facilitate systematic repurposing of off-patent medicines. |
| 16  A | Drug repurposing for rare: progress and opportunities for the rare disease community | Jonker et al. (2024) | Review the current status and opportunities for drug repurposing in rare diseases. | Emphasizes collaboration, regulatory support, and patient engagement as key drivers of successful repurposing in rare disease contexts. |
| 17 GL | Repurposing medicines in the NHS in England | NHS England | Describe the NHS strategy for supporting drug repurposing in the UK healthcare system. | Outlines a structured national approach to identifying, evaluating, and integrating repurposed medicines into clinical practice. |
| 18  A | Pioneering government-sponsored drug repositioning collaborations: progress and learning | Frail et al. (2015) | Share experiences from early government-led drug repurposing initiatives. | Shows how public funding can enable collaboration and accelerate access to underused compounds, while highlighting progress on IP and data transparency. |
| 19 GL | 2024 - KCE Trials 2024 investigator-led call | KCE Belgium (2024) | Announce funding for investigator-led clinical trials, including those on drug repurposing. | Supports early-stage clinical trials aimed at public health benefit, including innovative approaches to repurposing. |
| 20 GL | Repurposing Social Impact Bonds for Medicine | Stanford Social Innovation Review (2016) | Explore the use of social impact bonds to fund drug repurposing efforts. | Proposes an alternative financial mechanism to incentivise repurposing projects targeting unmet medical needs. |
| 21  A | Exploring new uses for existing drugs: innovative mechanisms to fund independent clinical research | Verbaanderd et al. (2021) | Identify innovative funding models to support non-commercial clinical research in drug repurposing. | Highlights mechanisms like public grants, crowdfunding, and hybrid models as promising tools for repurposing support. |
| 22 GL | Using Interventional Pharmacoeconomic Clinical Trials and Outcomes-Based Contracts to Repurpose Generic Drugs | Kerdemelidis (2024) | Advocate for pharmacoeconomic and outcomes-based models to support generic drug repurposing. | Suggests that aligning clinical and financial incentives can facilitate broader adoption of repurposed therapies. |
| 23 GL | Payer Funding Of Interventional Pharmacoeconomic Studies: A New Paradigm | Goldstein et al. (2019) | Propose payer-funded studies as a new paradigm for supporting drug repurposing. | Argues that involving any payers directly in funding can accelerate uptake of cost-effective repurposed treatments. |
| 24  A | Orphan/rare drug discovery through drug repositioning | Muthyala (2011) | Discuss drug repurposing as a method for orphan drug discovery. | Supports repurposing as a fast-track solution for neglected or rare disease treatments. |
| 25  A | Repurposing Approved and Abandoned Drugs for the Treatment and Prevention of Cancer | Weir et al. (2012) | Discuss public-private partnership strategies to repurpose medicines for oncology. | Highlights success cases carried out by the PPP *The Learning Collaborative* where repurposed drugs have been advanced through collaborative models in blood cancer care. |

Source: Own elaboration

Abbreviations: A=Academic paper; GL=Grey Literature paper
