# Supplementary material for: Overcoming barriers to off-patent drug repurposing: a lifecycle-based policy solutions
Source: Front Pharmacol. 2025 Oct 24;16:1670845. doi: 10.3389/fphar.2025.1670845 (PMC12592109; doi:10.3389/fphar.2025.1670845)
Supplement: Supplementary file 3 [file DataSheet5.docx]

SUPPLEMENTARY MATERIAL 5: Categorical qualitative analysis

| Categories | Questions | General Results | Sector | Involvement | Category Specialists |
| --- | --- | --- | --- | --- | --- |
| Challenges | *Current challenges* | Support for AcaRes/NGOs (60%) and the private initiative (56%), together with greater direct public funding (56%) and the adaptation of regulatory procedures (56%) | NRR | Most Involved Experts (MIE) advocate for incentives for the private sector (75%). Adapting regulatory procedures was not relevant by MIE (75%) | NRR |
| Research | *Opportunities identification* | Evidence from off-label use (91%) and RWE studies (77%) | Excluding Hospital Managers (HM) (0%), 75% of the rest sectors considered computational models | NRR | Computational models was strongest among preclinical experts. Non-preclinical experts supported the use of off-label (100%). |
|  | *Probability of success* (15/25 respondents) | Less than 30% (73.3%) | NRR | NRR | Clinical-stage experts agreed (100%) |
| R&D Funding for repurposing | R&D Funding problems | Lack of incentives for the private sector (60%), lack of public funding (56%), support for AcaRes/NGOs (56%) and difficulty of recovering R&D costs (56%) | 100% of Policymakers (PM) observed the general results too. Academic Researchers (AcaRes) highlighted the lack of public funding and support for AcaRes/NGOs | MIE highlighted the lack of private incentives (75%) | NRR |
|  | *Knowledge of R&D funding cases* (14/25) | Public funding (57%) | 80% AcaRes and Non-Governmental Organisations Professionals (NGOP) found any funding mechanisms | NRR | NRR |
|  | *R&D Funding Mechanisms* (19/25) | Public funding mechanisms (74%), PPPs (47%) | AcaRes and NGOs knew public funding mechanisms (100%) while Pharmaceutical Industry Professionals (PIP) and HM were more familiar with PPPs | Crowdfunding was only mentioned by the MIE (50%) | R&D Funding specialists knew PPPs (100%), Crowdfunding (60%) and (the only specialist group) impact investor funding with NGOs (40%) |
|  | *Requirements for R&D funding* (17/25) | Scientific criteria (76%), economic viability (71%), probability of success (71%) and consideration of public health need (59%) | Scientific, economic and commercial criteria were strongly supported by AcaRes (87.5%) | NRR | NRR |
| Regulatory aspects | *Regulatory challenges for AcaRes/NGOs* | Lack of knowledge of the process (88%) | HM pointed to a lack of public support (100%). PM and Agency Regulators (AR) highlighted the lack of procedures to facilitate robust clinical studies (100%) | Ocassional Involved Experts (OIE) pointed to low public support (69%) and MIE pointed to low involvement of the pharmaceutical industry (64%) | NRR |
|  | *Regulatory support programmes for AcaRes/NGOs* | 84% were aware at least one AcaRes/NGOs support programme. 72% knew the European "Repurposing of Authorised Medicines" programme | If HM are excluded, 94% of the rest sectors knew any programme. AcaRes, NGOP and AR knew the European Programme (100%) | NRR | NRR |
|  | *Boosting of Regulatory support programmes for AcaRes/NGOs* (16/25) | Limited effect (63%). Some contribution to boost R&D (87.5%) | NRR | NRR | NRR |
|  | *Knowledge of regulatory requirements* | Phase 3 clinical trial (83%) | NRR | Only the MIE (57%) saw off-label use studies | NRR |
|  | *Permissiveness in the requirements for regulatory approval* | Regulators should be more flexible in the requirements for regulatory approval (32%) | NRR | NRR | NRR |
|  | *Regulatory priority for particular situations* | Unmet health needs (72%) | AcaRes and HM support the main general result (93%) | NRR | Only 58% of regulatory specialists support the main result |
| Data Exclusivity | *Proposal for 4 years of data exclusivity as an incentive for repurposing* | It’s an effective incentive (67%) | NRR | MIE agreed with the main general result (82%) | Price-financing specialists agreed with the main general result (80%) |
| Role of public bodies | *Role of public bodies* | Regulatory support to AcaRes/NGOs (78%), R&D Funding (70%), identify new opportunities (65%) and foster PPPs (61%) | NRR | NRR | Public bodies specialists identify PPPs (85%) |
|  | *Sufficient funding from public bodies* | Insufficient public funding (90%) | NRR | NRR | Public bodies specialists support the main general result (100%) |
|  | *Increase in public investment* | Public investment should play a more active role (81%) | The main general result wasn’t shared by any PIP, while 94% of AcaRes, NGOP, PM and HM agreed with it. | NRR | NRR |

Source: Own elaboration

Abbreviations: NRR= No Relevant Results, PPPs= Public-Private Partnerships, AcaRes/NGOs= group of Academic Researchers and Non-Governmental Organization, RWE= Real-World Evidence
