# Supplementary material for: Overcoming barriers to off-patent drug repurposing: a lifecycle-based policy solutions
Source: Front Pharmacol. 2025 Oct 24;16:1670845. doi: 10.3389/fphar.2025.1670845 (PMC12592109; doi:10.3389/fphar.2025.1670845)
Supplement: Supplementary file 4 [file DataSheet2.docx]

DRUG REPURPOSING LIFE CYCLE QUESTIONNAIRE

*Estimated duration: 10-20 minutes*

The research we are working on is part of the **Horizon Europe** Health Innovation Next Generation Payment & Pricing Models (**HI-PRIX)** project. The overall objective of HI-PRIX is to find **mechanisms for public participation in the R&D process that allow fairer prices or cost savings for Health Systems**. Our work focuses on **drug repurposing** of off-patent medicines and how to identify efficient public sector strategies that can bring benefits to patients or cost savings to health systems.

In this case, we aim to gather information on **real cases** and current situation that will allow us to obtain results on the reality of repurposing projects for off-patent medicines.

Thus, we are writing to you with questions of interest to our research related to your **ACTIONS IN DRUG REPURPOSING**. In this way, the following questions address regulatory aspects, R&D funding, funding and market access for repurposed drugs and the role of the public sector.

Your name will appear in the **list of people consulted**, but the response will not be attributed to you. If you do not wish to appear in the list of people consulted, please **let us know** and only your professional profile will appear.

**Personal information**

- Name:
- Surname:
- Gender:
  1. Male
  2. Female
  3. I prefer not to say
- Age Range:
  1. 26-35
  2. 36-45
  3. 46-55
  4. 56-65
  5. +65
  6. I prefer not to say
- Years of experience in the sector or area:
  1. 0-5
  2. 5-10
  3. 10-20
  4. 30-40
  5. +40
- Sector of professional activity:
  1. Pharmaceutical industry
  2. Academic research
  3. Non-profit health-related organisation
  4. Related responsible in hospital
  5. Regulatory Agency
  6. Policymaker (Public Administration)
  7. Other: _______

*Name your position*

**Concept: Drug Repurposing**

1. Have you worked with or are you aware of the repurposing of **off-patent** medicines?

Yes/No

*If appropriate, please explain your answer*

1. In which **area** have you worked or do you have knowledge of` drug repurposing'? (multiple answers allowed)
   1. Regulatory aspects
   2. R&D in pre-clinical phases
   3. R&D in clinical phases
   4. R&D funding
   5. Drug pricing and financing
   6. Role of public bodies

*If appropriate, please explain your answer*

1. What **involvement** do you have or have you had in drug repurposing?
   1. Punctual / Few
   2. Quite active / Very active
   3. I dedicate myself exclusively to it

*If appropriate, please explain your answer*

1. Please tell me what are the **most important current challenges** in Spain and internationally for drug repurposing in new off-patent indications (multiple answers allowed).
   1. Development of economic support and incentives for private initiative
   2. Increased direct public funding for R&D
   3. Increased support and financial incentives for non-profit organisations/academic researchers
   4. Increased availability and interoperability of real-world data
   5. Further progress in identifying opportunities through computational modelling and/or AI
   6. Increased availability of early scientific consultation from regulatory agencies for research groups
   7. Regulatory procedures adapted to the specificities of drug repurposing
   8. Application of price differentiation by indication
   9. Implementation of innovative financing mechanisms for public participation or non-profit organisations (social impact bonds, etc.
   10. Other: ______

*If appropriate, please explain your answer.*

**Research**

1. In what way(s) do you identify an **opportunity** to initiate a 'drug repurposing' research project? (multiple answers allowed)
   1. Computational models for molecule identification
   2. Evidence through off-label use
   3. Studies with Real World Evidence
   4. Opinions of experts and health professionals
   5. Other: _______
   6. Question not aplicable

*If appropriate, please explain your answer*

1. Does drug repurposing research tend to target certain **clinical areas** (e.g. cancer, rare diseases, etc.)?

No, it's general / Yes (write which ones)

1. Can you tell me, give or take a range, the **likelihood of success** in identifying a repurposing opportunity to be ultimately repurposed? Being successful, commercial authorisation of the repurposed drug.
   1. -10%
   2. 10%-30%
   3. 30%-50%
   4. 50%-70%
   5. +70%
   6. Question not aplicable

*If appropriate, please explain your answer*

**R&D funding for repurposing**

1. In practice, what are the main **problems** encountered when trying to fund a drug repurposing research project (multiple answers posible)
   1. Lack of incentives for private initiative
   2. Lack of public funding
   3. Lack of support for academic researchers/non-profit organisations
   4. Low visibility and awareness for the pharmaceutical community
   5. Difficulty in providing sufficient clinical evidence for regulatory requirements
   6. Difficulty in promoting successful results of candidate repositioning molecules obtained through computational modelling
   7. Difficulty in recovering the cost of R&D in the final price
   8. Other: ______

*If appropriate, please explain your answer.*

1. Have you found **R&D funding for** drug repurposing projects?
   1. Yes, private funding
   2. Yes, public funding
   3. Yes, funding from not-for-profit organisations
   4. Yes, another guy: _________
   5. No, none
   6. Question not aplicable

*If appropriate, please explain your answer.*

1. Do you know of any real-world examples of **R&D funding mechanisms** where funding has been obtained by academic researchers or non-profit organisations to fund 'drug repurposing' multiple answers allowed)?
   1. Funding from public health agencies or programmes
   2. Crowdfunding
   3. Commercial party acquisition by highly reputable researchers
   4. Impact investors (Venture capital) in collaborations with health non-profits
   5. Public-private partnerships
   6. Social Impact Bonds
   7. Other: _____

*If appropriate, please explain your answer*

1. Given your experience, what **requirements** does the funder ask for in order to approach the project (multiple answers allowed)?
   1. Economic viability
   2. Scientific criteria
   3. Probability of marketing success
   4. Consideration of public health need
   5. Other: _______
   6. Question not aplicable

*If appropriate, please explain your answer*

**Regulatory aspects of repurposing**

1. What challenges do **academic researchers and/or non-profit organisations** face when dealing with regulatory approval of a drug repurposing project? (multiple answers allowed)
   1. Lack of public support
   2. Lack of knowledge of the regulatory process
   3. Lack of procedures for robust clinical studies
   4. Lack of awareness of the peculiarities of 'drug repurposing'.
   5. Lack of involvement of the pharmaceutical industry
   6. Other: ________

*If appropriate, please explain your answer*

1. Are you aware of any of the following **programmes supporting** academic researchers/non-profit organisations **for regulatory approval** of a repurposed drug?
   1. Repurposing Observatory Group (STAMP Expert Group)
   2. UK Innovative Licensing and Access Pathway
   3. Discovering New Therapeutic Uses for Existing Molecules (United States)
   4. Repurposing of Authorised Medicines (EU)
   5. Innovation and Knowledge Support Office (Spain)
   6. Other: _______
   7. I don't know of any

*If appropriate, please explain your answer*

1. If you are aware of any programmes supporting academic researchers/non-profit organisations for regulatory approval, are they succeeding in **boosting** drug repurposing R&D?
   1. Nothing
   2. Significantly little
   3. There has been a moderately significant change
   4. There has been a very significant empowerment
   5. Question not aplicable

*If appropriate, please explain your answer*

1. In a real case of 'drug repurposing' that you are aware of, what were the **requirements** that regulators requested in order to grant marketing authorisation to repurpose a medicine (multiple answers allowed)?
   1. Random EC phase 3
   2. Random EC phase 1 or 2
   3. Safety data and adverse effects in the new indication
   4. Studies based on real world evidence (no RCTs)
   5. Studies based on off-label use
   6. Question not aplicable

*If appropriate, please explain your answer*

1. Should regulators have been more **lenient** in their marketing authorisation requirements for repositioning a medicine based on real-life experiences?
   1. Strongly disagree
   2. Somewhat disagree
   3. They are correct
   4. Somewhat in agreement
   5. I fully agree

*If appropriate, please explain your answer*

1. Do you think that new 'drug repurposing' indications for some special situations should be **prioritised** for regulatory approval (multiple answers allowed)?
   1. Unmet health needs
   2. Treatments that do not cure, but serve to alleviate symptoms
   3. Treatments to improve quality of life
   4. They should not have different criteria than other medicines
   5. Certain clinical areas: __________
   6. Other: _______

*Express your opinion*

**Price and funding of the repositioned medicine**

*SUPPORTING INFORMATION ON ARTICLE 84 OF THE NEW EU LEGISLATION*:

Data protection for repurposed medicines

A regulatory data protection period of four years shall be granted for a medicinal product in respect of a new therapeutic indication not previously authorised in the Union, provided that:

(a) adequate clinical or non-clinical studies have been conducted in relation to the therapeutic indication demonstrating that it is of significant clinical benefit, and

(b) the medicinal product is authorised in accordance with Articles 9 to 12 and has not previously benefited from data protection, or 25 years have elapsed since the granting of the initial marketing authorisation for the medicinal product concerned.

The period of data protection referred to in paragraph 1 may be granted only once for a given medicinal product. During the period of data protection referred to in paragraph 1, the marketing authorisation shall state that the medicinal product is an existing medicinal product authorised in the Union with an additional therapeutic indication.

1. Can the proposal in the new European pharmaceutical legislation for a **4-year** period of **data exclusivity** ([Art 84](https://health.ec.europa.eu/medicinal-products/eudralex/eudralex-volume-1_en#:~:text=Today%20the%20EU%20legal%20framework%20for%20medicinal%20products,market%20with%20measures%20that%20encourage%20innovation%20and%20competiveness.)) for a repositioned medicine be a valuable incentive for R&D?
   1. No
   2. Yes, but 4 years of data exclusivity is not enough.
   3. Yes, it can be a valuable incentive.
   4. Yes, it is a great incentive

*If appropriate, please explain your answer*

1. If data protection may exist for a repositioned indication, how can the fact that **the indication** that is protected with a higher price is **differentiated from the generic** with the original indication be enforced?
   1. It is not possible to differentiate the price per indication
   2. It is possible to differentiate the price per indication

*Explain your answer*

**The role of public bodies in drug repurposing**

1. What is the current role of the **public sector** from the financing of drug repurposing projects to their subsequent access to the market (multiple answers possible)?
   1. Identification of drug opportunities to be repositioned
   2. Funding of drug R&D through any funding mechanism for public participation
   3. Regulatory support for academic researchers/non-profit organisations
   4. Drug financing
   5. Public-private partnerships
   6. Other: _______

*If appropriate, please explain your answer*

1. Is funding from **public agencies** sufficient for drug repurposing projects?
   1. No, it is tiny
   2. No, some more effort is needed
   3. Yes, but it is poorly managed
   4. Yes, it is sufficient, the public body should not have to make any further financial effort.

*If appropriate, please explain your answer*

1. Do you know of any cases of 'drug repurposing' in which there has been significant **public involvement** in its financing?

No/Yes (explain)

1. Should **public investment** play a more prominent role in funding drug repurposing projects?
   1. Strongly disagree
   2. Somewhat disagree
   3. They are correct
   4. Somewhat in agreement
   5. I fully agree

*If appropriate, please explain your answer*

**Open questions from real cases**

1. If it has not been explained in previous questions, could you explain any **successful** experience in drug repurposing?
2. If it has not been explained in previous questions, could you explain any **unsuccessful** experiences in drug repurposing?
3. What factors are decisive for the **success of a** drug repurposing project?

*Finally, would you be interested in providing further information on the subject by conducting an interview with the researchers?*

Yes/no

CUESTIONARIO SOBRE EL CICLO DE VIDA DEL ‘DRUG REPURPOSING’

*Duración estimada: 10-20 minutos*

La investigación sobre la que trabajamos está encuadrada en el proyecto Health Innovation Next Generation Payment & Pricing Models (**HI-PRIX)** del plan **Horizon Europe**. El objetivo general de HI-PRIX es encontrar **mecanismos de participación pública en el proceso de I+D que permitan precios más justos o ahorros de costes para los Sistemas de Salud**. Nuestro trabajo se centra en el reposicionamiento de medicamentos (‘**drug repurposing’**) que se encuentran fuera de patente y en cómo identificar estrategias eficientes del sector público que puedan aportar beneficios a los pacientes o ahorrar costes a los sistemas sanitarios.

En este caso, nos dirigimos a recopilar información sobre **casos reales** y situación actual que nos permita obtener resultados sobre la realidad de los proyectos de ‘repurposing’ de medicamentos que están fuera de patente

Así, nos dirigimos a usted con preguntas de interés para nuestra investigación relacionadas por sus **ACTUACIONES EN EL ’DRUG REPURPOSING’**. De esta manera, las siguientes preguntas responden a aspectos regulatorios, financiación de I+D, financiación y acceso al mercado de ’repurposed drugs’ y el papel del sector público.

Su nombre aparecerá en el **listado de personas consultadas**, pero no se le atribuirá la respuesta. Si usted desea que no aparezca en el listado de personas consultadas, **comuníquelo** y sólo aparecerá su perfil profesional.

**Informacion personal**

- Nombre:
- Apellidos:
- Género:
  1. Masculino
  2. Femenino
  3. Prefiero no decirlo
- Intervalo Edad:

1. 26-35
2. 36-45
3. 46-55
4. 56-65
5. +65
6. Prefiero no decirlo

- Años de experiencia en el sector o área:

1. 0-5
2. 5-10
3. 10-20
4. 30-40
5. +40

- Sector de actividad profesional:

1. Industria Farmacéutica
2. Investigación académica
3. Organización sin ánimo de lucro relacionada con la salud
4. Responsable relacionado en hospital
5. Agencia reguladora
6. Policymaker (Administración Pública)
7. Otro: _______

*Nombre su puesto*

**Concepto: Drug Repurposing**

1. ¿Ha trabajado o tiene conocimiento acerca del ‘repurposing’ de medicamentos que se encuentran **fuera de patente**?

Sí/No

*Si lo ve conveniente, explique su respuesta*

1. ¿En qué **área** ha trabajado o tiene conocimiento usted sobre el `drug repurposing’? (se admiten respuestas múltiples)
2. Aspectos regulatorios
3. I+D en fases preclínicas
4. I+D en fases clínicas
5. Financiación de I+D
6. Precio y financiación del medicamento
7. Papel de los organismos públicos

*Si lo ve conveniente, explique su respuesta*

1. ¿Qué **implicación** tiene o ha tenido usted en el ’drug repurposing’?
2. Puntual / Poca
3. Bastante / Mucha actividad
4. Me dedico exclusivamente a ello

*Si lo ve conveniente, explique su respuesta*

1. Cuénteme, por favor, cuáles son los **retos actuales más importantes** en España y a nivel internacional para el ‘drug repurposing’ en nuevas indicaciones de medicamentos fuera de patente. (se admiten respuestas múltiples)
2. Desarrollo de apoyo e incentivos económicos para la iniciativa privada
3. Mayor financiación pública directa a I+D
4. Mayor apoyo e incentivos económicos a organizaciones sin ánimo de lucro/investigadores académicos
5. Mayor disponibilidad e interoperabilidad de datos del mundo real
6. Mayor avance en la identificación de oportunidades a través de modelos computacionales y/o IA
7. Mayor disponibilidad de consulta científica temprana de las agencias regulatorias para grupos de investigación
8. Procedimientos regulatorios adaptados a las peculiaridades del ‘drug repurposing’
9. Aplicación de diferenciación de precios por indicación
10. Aplicación de mecanismos innovadores de financiación de participación pública u organizaciones sin ánimo de lucro (social impact bonds, etc.)
11. Otro: ______

*Si lo ve conveniente, explique su respuesta.*

**Investigación**

1. ¿De qué manera(s) se identifica una **oportunidad** para iniciar un proyecto de investigación de ‘drug repurposing’? (se admiten respuestas múltiples)
2. Modelos computacionales de identificación de moléculas
3. Evidencias a través del uso off-label
4. Estudios con Real World Evidence
5. Opinión de expertos y profesionales sanitarios
6. Otro: _______
7. Pregunta no aplicable

*Si lo ve conveniente, explique su respuesta*

1. ¿Las investigaciones de ‘drug repurposing’ se suelen dirigir a ciertas **áreas clínicas** (por ejemplo, cáncer, enfermedades raras, etc.)?

No, es general / Sí (escribe cuáles)

1. ¿Sabría decirme, más o menos un rango, la **probabilidad de éxito** que tiene la identificación de una oportunidad de ’repurposing’ para ser finalmente ‘repurposed’? Siendo éxito, la autorización comercial del ’repurposed drug’.
2. -10%
3. 10%-30%
4. 30%-50%
5. 50%-70%
6. +70%
7. Pregunta no aplicable

*Si lo ve conveniente, explique su respuesta*

**Financiación de I+D para reposicionamiento**

1. En la práctica, ¿Cuáles son los principales **problemas** que se encuentran cuando se quiere financiar un proyecto de investigación de ‘drug repurposing’? (se admiten múltiples respuestas)
2. Falta de incentivos para la iniciativa privada
3. Falta de financiación pública
4. Falta de apoyo para los investigadores académicos/organizaciones sin ánimo de lucro
5. Poca visibilidad y concienciación para la comunidad farmacéutica
6. Dificultad de disponer de evidencia clínica suficiente para las exigencias regulatorias
7. Dificultad de promoción de resultados exitosos de moléculas candidatas a reposicionar obtenidos a través de modelos computacionales
8. Dificultad de recuperar el coste de la I+D en el precio final
9. Otro: ______

*Si lo ve conveniente, explique su respuesta.*

1. ¿Se ha encontrado usted **financiación para el I+D** de proyectos de ’drug repurposing’?
2. Sí, financiación privada
3. Sí, financiación pública
4. Sí, financiación a partir de organismos sin ánimo de lucro
5. Sí, otro tipo: _________
6. No, ninguna
7. Pregunta no aplicable

*Si lo ve conveniente, explique su respuesta.*

1. ¿Conoce usted ejemplos, en el mundo real, de **mecanismos de financiación de I+D**, cuya financiación haya sido obtenida por investigadores académicos u organizaciones sin ánimo de lucro para financiar ‘drug repurposing’? (se admiten respuestas múltiples)
2. Financiación a partir de agencias o programas públicos de salud
3. ‘Crowdfunding’
4. Captación de partes comerciales por parte de investigadores de alta reputación
5. Inversores de impacto (Venture capital) en colaboraciones con organizaciones sin ánimo de lucro de salud
6. Colaboración público-privada
7. Social Impact Bonds
8. Otro: _____

*Si lo ve conveniente, explique su respuesta*

1. Teniendo en cuenta su experiencia, ¿qué **requisitos** pide el financiador para abordar el proyecto? (se admiten respuestas múltiples)
2. Viabilidad económica
3. Criterios científicos
4. Probabilidad de éxito de comercialización
5. Consideración de necesidad de salud pública
6. Otro: _______
7. Pregunta no aplicable

*Si lo ve conveniente, explique su respuesta*

**Aspectos regulatorios de ‘drug repurposing’**

1. ¿Qué retos tienen los **investigadores académicos y/o las organizaciones sin ánimo de lucro** a la hora de enfrentarse a la aprobación regulatoria de un proyecto de ’drug repurposing’? (se admiten respuestas múltiples)
2. Falta de apoyo público
3. Falta de conocimiento del proceso regulatorio
4. Falta de procedimientos para realizar estudios clínicos robustos
5. Falta de concienciación respecto a las peculiaridades del ‘drug repurposing’
6. Falta de participación de la industria farmacéutica
7. Otro: ________

*Si lo ve conveniente, explique su respuesta*

1. ¿Conoce alguno de los siguientes **programas de apoyo** a investigadores académicos/organizaciones sin ánimo de lucro **para la aprobación regulatoria** de un ‘repurposed drug’?
2. Repurposing Observatory Group (Grupo experto STAMP)
3. UK Innovative Licensing and Access Pathway
4. Discovering New Therapeutic Uses for Existing Molecules (United States)
5. Repurposing of Authorized Medicines (UE)
6. Oficina de Apoyo a la Innovación y Conocimiento (España)
7. Otro: _______
8. No conozco ninguno

*Si lo ve conveniente, explique su respuesta*

1. Si conoce algún programa de apoyo a investigadores académicos/organizaciones sin ánimo de lucro para la aprobación regulatoria, ¿están consiguiendo **potenciar** la I+D del ’drug repurposing’?
2. Nada
3. Significativamento poco
4. Ha habido un cambio medianamente significativo
5. Ha habido una potenciación muy significativa
6. Pregunta no aplicable

*Si lo ve conveniente, explique su respuesta*

1. ¿En un caso real de ’drug repurposing’ que usted conoce, ¿Cuáles fueron los **requisitos** que solicitaron los reguladores para conceder la autorización comercial para reposicionar un medicamento? (se admiten respuestas múltiples)
2. EC aleatorio fase 3
3. EC aleatorio fase 1 o 2
4. Datos de seguridad y efectos adversos en la nueva indicación
5. Estudios basados en real world evidence (sin ECA)
6. Estudios basados en uso off-label
7. Pregunta no aplicable

*Si lo ve conveniente, explique su respuesta*

1. Teniendo en cuenta experiencias en casos reales ¿Deberían haber sido más **permisivos** los reguladores en las exigencias para la autorización comercial para reposicionar un medicamento?
2. Totalmente en desacuerdo
3. Un poco en desacuerdo
4. Son correctas
5. Un poco de acuerdo
6. Totalmente de acuerdo

*Si lo ve conveniente, explique su respuesta*

1. ¿Piensa que deben tener **prioridad** para la aprobación regulatoria las nuevas indicaciones de ‘drug repurposing’ para algunas situaciones especiales? (se admiten respuestas múltiples)
2. Necesidades de salud no cubiertas
3. Tratamientos no curativos, pero que sirven para paliar síntomas
4. Tratamientos para mejorar la calidad de vida
5. No deben tener criterios distintos a otros medicamentos
6. Ciertas áreas clínicas: __________
7. Otro: _______

*Exprese su opinión*

**Precio y financiación del medicamento reposicionado**

INFORMACIÓN DE APOYO SOBRE EL ARTÍCULO 84 DE LA NUEVA LEGISLACIÓN EUROPEA:

Protección de datos para medicamentos readaptados

Se concederá un período de protección normativa de datos de cuatro años para un medicamento con respecto a una nueva indicación terapéutica no autorizada previamente en la Unión, siempre que:

a) se hayan llevado a cabo estudios clínicos o no clínicos adecuados en relación con la indicación terapéutica que demuestren que presenta un beneficio clínico significativo, y

b) el medicamento esté autorizado de conformidad con los artículos 9 a 12 y no se haya beneficiado previamente de la protección de datos, o hayan transcurrido veinticinco años desde la concesión de la autorización inicial de comercialización del medicamento en cuestión.

El período de protección de los datos a que se refiere el apartado 1 solo podrá concederse una vez para un medicamento determinado. Durante el período de protección de los datos a que se refiere el apartado 1, la autorización de comercialización indicará que el medicamento es un medicamento existente autorizado en la Unión con una indicación terapéutica adicional.

1. ¿Puede ser la propuesta en la nueva legislación farmacéutica europea para un periodo de **4 años de exclusividad de datos** ([Art 84](https://health.ec.europa.eu/medicinal-products/eudralex/eudralex-volume-1_en#:~:text=Today%20the%20EU%20legal%20framework%20for%20medicinal%20products,market%20with%20measures%20that%20encourage%20innovation%20and%20competiveness.)) para un medicamento reposicionado un incentivo valorable para I+D?
2. No
3. Sí, pero 4 años de exclusividad de datos no es suficiente
4. Sí, puede ser un incentivo bastante valorable
5. Sí, es un gran incentivo

*Si lo ve conveniente, explique su respuesta*

1. En caso de que pueda existir una protección de datos para una indicación reposicionada, ¿cómo se puede cumplir el hecho de que se **diferencie la indicación** que está protegida con un precio mayor **respecto del genérico** con la indicación original?
2. No es posible diferenciar el precio por indicación
3. Sí es posible diferenciar el precio por indicación

*Explique su respuesta*

**Papel de los organismos públicos en el ’drug repurpoisng’**

1. ¿Cuál es el papel actual del **sector público** desde la financiación de proyectos de ‘drug repurposing’ hasta su posterior acceso al mercado? (se admiten respuestas múltiples)
2. Identificación de oportunidades de fármacos a reposicionar
3. Financiación de la I+D del medicamento a través de cualquier mecanismo de financiación de participación pública
4. Apoyo regulatorio a investigadores académicos/organizaciones sin ánimo de lucro
5. Financiación del medicamento
6. Colaboración público-privada
7. Otro: _______

*Si lo ve conveniente, explique su respuesta*

1. ¿Es suficiente la financiación de los **organismos públicos** para proyectos de ’drug repurposing’?
2. No, es ínfima
3. No, se necesita algún esfuerzo más
4. Sí, pero está mal gestionada
5. Sí, es suficiente, el organismo público no debe de realizar mayor esfuerzo financiero

*Si lo ve conveniente, explique su respuesta*

1. ¿Conoce algún caso de ’drug repurposing’ en el que haya existido una importante **participación pública** en su financiación?

No/Sí (explíquelo)

1. ¿La **inversión pública** debería ser más protagonista a la hora de financiar proyectos de ’drug repurposing’?
2. Totalmente en desacuerdo
3. Un poco en desacuerdo
4. Son correctos
5. Un poco de acuerdo
6. Totalmente de acuerdo

*Si lo ve conveniente, explique su respuesta*

**Preguntas abiertas de casos reales**

1. Si no ha sido explicado en preguntas anteriores, ¿podría explicar alguna experiencia **exitosa** en un ’drug repurposing’?
2. Si no ha sido explicado en preguntas anteriores, ¿podría explicar alguna experiencia **no exitosa** en un ’drug repurposing’?
3. ¿Qué factores son determinantes para el **éxito** de un proyecto de ‘drug repurposing’?

*Por último, ¿estaría interesado/a en aportar más declaraciones sobre el tema a través de una realización de una entrevista con los investigadores?*

Sí/No
