# Supplementary material for: Overcoming barriers to off-patent drug repurposing: a lifecycle-based policy solutions
Source: Front Pharmacol. 2025 Oct 24;16:1670845. doi: 10.3389/fphar.2025.1670845 (PMC12592109; doi:10.3389/fphar.2025.1670845)
Supplement: Supplementary file 5 [file DataSheet1.docx]

SUPPLEMENTARY MATERIAL 1: Search strategy and flow chart diagram

*Academic search strategy on PubMed and Embase:*

- The search lines were:

1. **Repurposing**

Repurposing OR repurpose* OR repositioning OR “new uses”

1. **Drug**

Drug* OR medicine* OR pharmaceutical

1. **Target medicines**

Generic OR off-patent OR biosimilar OR “existing medicines” OR “existing drugs” OR “abandoned drugs” OR “authorized medicines” OR rare OR orphan OR cancer OR anticancer

1. **R&D**

Research OR “R&D” OR Development

1. **Regulatory process**

Regulator*

1. **Price and financing**

Fund* OR financing OR pricing OR price

1. **Non-profit researchers**

“Non-profit” OR “not-for-profit” OR NGO OR academi*

1. **Public sector**

Government OR “public sector”

1. **Policy**

Model* OR program* OR incentives OR mechanism* OR contract* OR policy OR policies OR legal OR legislation

1. **Actuality**

Challenges OR Opportunities OR progress

- The search strategy was:

[#1 AND #2] AND [#3 OR #4 OR #5 OR #6 OR #7 OR #8 OR #9 OR #10]

*Flowchart diagram:*


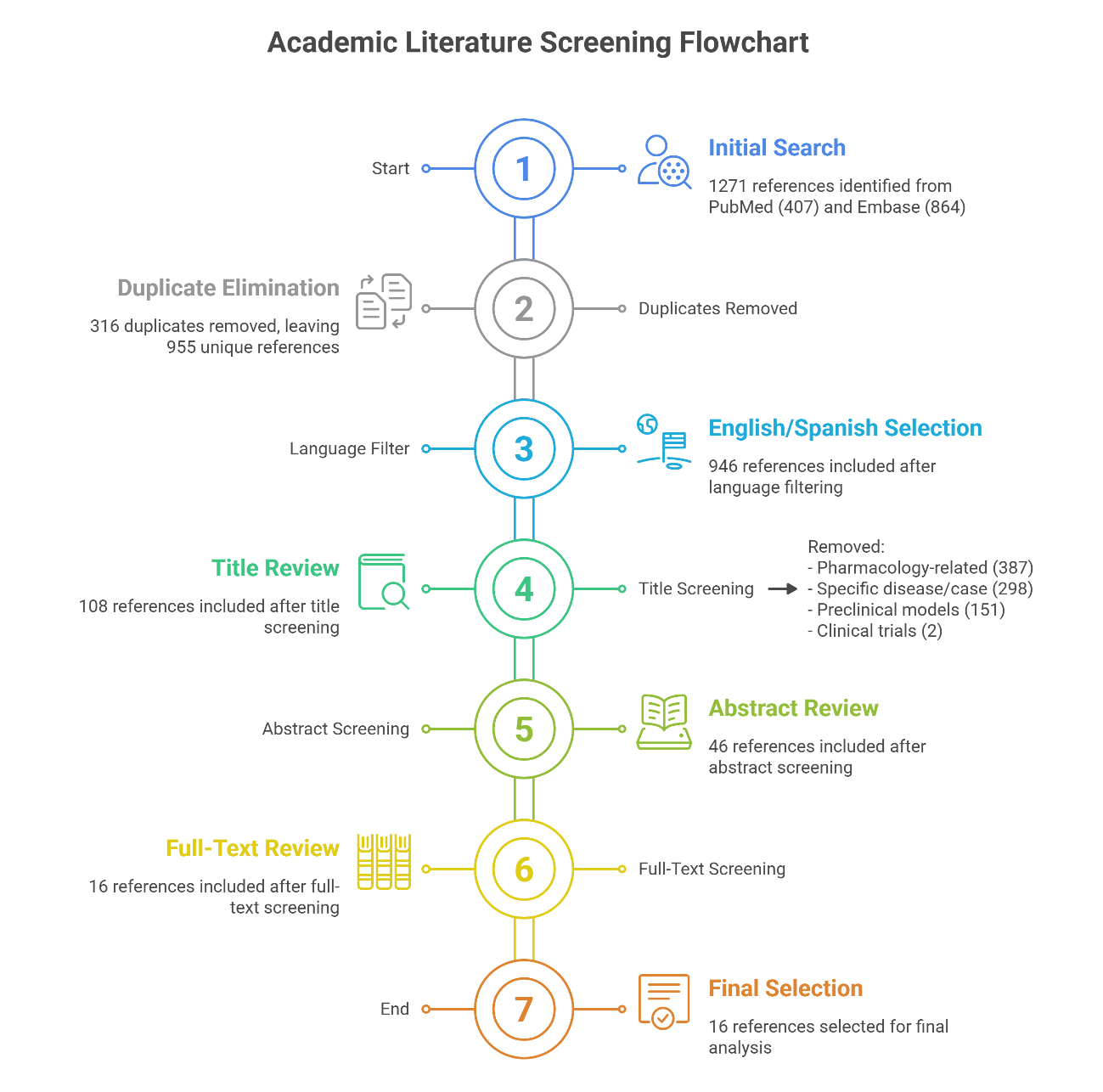


*Grey literature search strategy:*

1. **Search:**

- For public intervention mechanisms, the keywords used were:

"drug repurposing" AND (generic OR off-patent OR financing OR pricing OR incentives)

- For government-sponsored collaborative drug repurposing projects, the keywords used were:

"drug repurposing" AND (government OR policy OR public OR NGO OR academia)

1. **Final Selection:**

- For public intervention mechanisms,

5 grey literature articles selected

- For government-sponsored collaborative drug repurposing projects,

4 grey literature articles selected
